# ShEnrich: a functional annotation database and enrichment platform for penaeid shrimp

**DOI:** 10.1186/s12859-026-06567-0

**Published:** 2026-08-03

**Authors:** Harikrishnan Udayakumar Geetha, Ashok Kumar Jangam, Vinaya Kumar Katneni, Syama Dayal Jagabattula, Panjan Nathamuni Suganya, Mudagandur Shashi Shekhar, Ritwika Das, Monendra Grover, Girish Kumar Jha

**Affiliations:** 1 Nutrition Genetics and Biotechnology Division, ICAR-Central Institute of Brackishwater Aquaculture, Chennai, 600028 India; 2 Aquatic Animal Health and Environment Division, ICAR- Central Institute of Brackishwater Aquaculture, Chennai, 600028 India; 3 Agriculture Bioinformatics Division, ICAR-Indian Agricultural Statistics Research Institute, New Delhi, India

**Keywords:** Penaeid shrimp, Functional annotation, Gene ontology, KEGG pathway enrichment, Comparative genomics, Aquaculture genomics, Non-model organisms, Transcriptomics, Ortholog clustering

## Abstract

**Background:**

Penaeid shrimp are among the most commercially important aquaculture species globally, yet no dedicated integrated functional annotation and enrichment analysis platform currently for penaeid shrimp. Researchers working with shrimp transcriptomic data currently rely on generic enrichment tools built around model organisms that provide limited or no coverage for most penaeid species. In this context, current study aims to develop annotation and enrichment analysis platform for commercially important shrimp species.

**Methods:**

ShEnrich was developed using a multi-tiered annotation pipeline combining BLASTP searches against the NCBI non-redundant database, InterProScan domain prediction, and eggNOG-mapper orthology assignments. Cross-species ortholog clustering was performed using OrthoMCL across five commercially farmed penaeid species. GMT libraries for each species were constructed for KEGG and GO enrichment analysis, implemented using the clusterProfiler R package. The platform was built on a MySQL 8.0 relational database with a PHP 8.1 backend and an integrated JBrowse2 genome browser.

**Results:**

The database integrates 61,287 annotated proteins representing 34,169 genes, with 178,595 gene-pathway associations across 442 KEGG pathways and 138,077 proteins mapped to 12,897 GO terms across five species: *Penaeus vannamei*, *Penaeus monodon*, *Penaeus indicus*, *Penaeus chinensis*, and *Penaeus japonicus*. Cross-species comparative genomics is supported through 18,031 ortholog groups identified using OrthoMCL. The web platform provides KEGG and GO enrichment analysis, protein annotation search, ortholog browsing, and genome visualization. ShEnrich is freely available at https://bioinfo.ciba.res.in/shenrich/.

**Conclusions:**

ShEnrich provides the first dedicated functional annotation and enrichment analysis resource for penaeid shrimp, filling a gap that has limited biological interpretation of transcriptomic data in this economically important group. The platform supports functional analysis of gene expression studies across disease, stress, and environmental conditions in commercially farmed shrimp species.

## Background

High-throughput sequencing technologies have transformed aquaculture genomics, generating large volumes of transcriptomic data across multiple shrimp species and experimental conditions [1]. Translating these datasets into biological insights depends on accurate functional annotation of genome sequences, which forms the foundation for downstream analyses including Gene Ontology (GO) term enrichment and Kyoto Encyclopedia of Genes and Genomes (KEGG) pathway enrichment analysis [2]. While robust annotation databases exist for model organisms such as FlyBase for *Drosophila* and TAIR for *Arabidopsis* [3, 4], equivalent resources for aquaculture species are largely absent. Among aquaculture species, penaeid shrimp lack a dedicated integrated functional annotation and enrichment analysis platform, with dedicated genomic resources remaining limited compared to better-studied aquaculture groups such as salmonids [5] and catfish [6], while penaeid shrimp remain critically underserved despite being among the most widely farmed organisms globally.

Penaeid shrimp genomes have become available for several commercially important species in recent years [7–11], and the volume of RNA-seq studies in this group has grown considerably, covering responses to pathogens, environmental stressors, and nutritional conditions [12, 13]. However, functional annotation infrastructure has not kept pace with genome sequencing efforts. Existing enrichment tools such as DAVID, Enrichr, and pathDIP rely primarily on annotations derived from model organisms and offer limited coverage for crustaceans [14–16]. Tools such as getENRICH support enrichment analysis for non-model organisms through KO-based annotations [17], but curated species-specific gene sets and annotation pipelines for penaeid shrimp have not been reported in the literature. The reliance on distant ortholog projections results in incomplete functional assignments, with many crustacean-specific genes remaining unannotated or misannotated.

Penaeid shrimp possess specialized physiological mechanisms for osmoregulation, molting, immune defense, and stress responses that are distinct from those of model organisms [18, 19]. Some of the genes governing these processes have limited representation in model organism databases, and generic enrichment tools may not fully capture pathways relevant to crustacean physiology due to the under-representation of crustacean sequences in their background gene sets. A dedicated annotation resource for penaeid shrimp would therefore support more complete functional interpretation of transcriptomic data in this group, rather than relying solely on annotations projected from distantly related organisms [20, 21]. Therefore, the aim of this study is to build an easy-to-use annotation and enrichment analysis platform for penaeid shrimp species.

We developed ShEnrich, a curated relational database and enrichment analysis resource dedicated to five commercially important penaeid shrimp species: *Penaeus vannamei*, *Penaeus monodon*, *Penaeus chinensis*, *Penaeus indicus*, and *Penaeus japonicus*. ShEnrich provides species-specific functional annotations generated through a multi-tiered pipeline combining homology searches, protein domain prediction, and orthology-based inference. Built on this annotation database, the platform supports KEGG and GO-based enrichment analysis using curated species-specific gene sets, ortholog group identification across all five species, and genome visualization through an integrated browser. ShEnrich provides a unified interface combining functional annotation retrieval, enrichment analysis, ortholog browsing, and genome visualisation dedicated to penaeid shrimp.

## Construction and content

### Genome data acquisition

Genome assemblies and protein sequence datasets for five commercially important *Penaeus* species were obtained from NCBI RefSeq (accessed January, 2025) *- P. vannamei* (GCF_042767895.1; ASM4276789v1) [22], *P. monodon* (GCF_015228065.2; NSTDA_Pmon_1) [8], *P. chinensis* (GCF_019202785.1; ASM1920278v2) [9], *P. indicus* (GCF_018983055.1; CIBA_Pind_1.3) [11], and *P. japonicus* (GCF_017312705.1; Mj_TUMSAT_v1.0) [10]. Summary statistics for each assembly including genome size, number of contigs, scaffolds, N50, annotated proteins, KEGG pathways covered, and unique genes are provided in Table 1.

**Table 1:** Genome statistics of five shrimp species in ShEnrich

Species	Size of genome (Gb)	Contigs	Scaffolds	N50 (Mb)	Annotated Proteins	KEGG Pathways	GO background	KEGG background
*P. monodon*	2.4	1,185,336	26,634	44.9	10,280	391	21,888	1,862
*P. vannamei*	1.8	20,004	5,443	42.3	15,183	435	35,459	14,448
*P. japonicus*	1.7	28,883	18,210	0.234	13,356	390	30,684	2,407
*P. chinensis*	1.5	9,042	1,060	36.9	11,862	338	27,250	2,097
*P. indicus*	1.9	18,853	10,969	34.6	10,606	340	28,137	2,056

Annotated Proteins: Unique number of proteins with KEGG pathway assignment. KEGG background: Total number of KEGG pathway assignments for the proteins used as background in KEGG enrichment analysis. GO background: Total number of GO term assignments for the proteins used as background in GO enrichment analysis

### Functional annotation pipeline

A comprehensive, multi-tiered annotation pipeline was implemented to maximize functional coverage of penaeid shrimp genes while prioritizing phylogenetically proximate annotations. Protein sequences from each species were subjected to BLASTP [23] against the NCBI non-redundant (nr) protein database, with an e-value threshold of 1 × 10⁻⁵. This approach prioritizes functional assignments from evolutionarily proximate species, though it is acknowledged that crustacean protein annotations in reference databases are themselves largely derived from experimentally characterized model organisms. Gene Ontology (GO) term assignments were obtained by integrating BLAST results with the GO database [24]. Parallel to homology-based annotation, domain-based functional prediction was conducted using InterProScan v5.52-86.0 [25], to identify conserved functional domains and motifs. Orthology-based annotation was conducted using eggNOG-mapper v2.1.9 [26] against the eggNOG 5.0 database, providing KEGG Orthology (KO) assignments, Orthologous functional categories, and additional GO term predictions based on orthologous gene clusters. Pathway annotations were generated by mapping KO assignments to KEGG pathways using KEGG database, resulting in annotations spanning 442 distinct pathway identifiers across 48 metabolism categories. All annotation outputs were consolidated into a unified annotation table for each species through the integrated Blast2GO consolidation step within OmicsBox [27], which integrates outputs from all three annotation tiers. Here, each GO term receives a composite annotation score based on cumulative support from BLASTP similarity, InterProScan domain evidence, and eggNOG-mapper orthology assignments, and terms with insufficient combined evidence are filtered out.

### Identification of orthologous gene clusters

To facilitate comparative genomics studies across the five *Penaeus* species, we performed comprehensive gene family clustering using OrthoMCL v2.0.9 [28] with default parameters. Non-redundant good protein sequences with a minimum length of 50 amino acids from all the five shrimp species were used as input. All-versus-all BLAST was conducted with an e-value cutoff of 1 × 10⁻⁵. The resulting similarity scores were normalized using the OrthoMCL algorithm to account for differences in protein length and sequence composition, then subjected to the Markov Cluster Algorithm (MCL) [29]. This analysis identified 18,031 ortholog groups encompassing genes from all the five *Penaeus* species. Each ortholog group represents a set of genes sharing common ancestry, enabling functional inference across species and identification of lineage-specific gene expansions or losses.

### Gene matrix transposed (GMT) library construction

To enable efficient enrichment analysis using standard tools while maintaining species-specific annotation accuracy, curated Gene Matrix Transposed (GMT) libraries were constructed for each *Penaeus* species, one for GO terms and other for KEGG pathways. Gene-to-GO and gene-to-pathway associations were extracted from the integrated annotation tables using custom Python scripts. Each GMT entry contains the pathway/GO identifier, description, and the complete list of gene accession IDs formatted according to standard GMT specifications [30]. These curated GMT files were utilized for species-specific enrichment analyses within ShEnrich, enabling pathway-level annotations.

### Enrichment analysis implementation

ShEnrich provides two main enrichment analysis modules based on established methodologies from the clusterProfiler R package (v4.17.0) [31, 32]. Both modules employ Fisher’s exact test to evaluate statistical over-representation of functional categories within user-provided gene lists relative to the species-specific background gene set. To perform enrichment analysis, users have to upload gene lists as protein accession identifiers, select the species, and specify statistical parameters such as significance threshold and multiple testing correction method.

The pathway enrichment module tests for over-representation of KEGG pathways within user-provided gene lists relative to the species-specific background gene set. The GO enrichment module evaluates over-representation across Biological Process, Cellular Component, and Molecular Function ontologies. Both tools support multiple testing correction options including Benjamini-Hochberg false discovery rate (FDR) correction [33], Bonferroni correction, and uncorrected P-values. Output visualizations including dot plots, bar charts, and gene-concept network diagrams were generated using the open-source JavaScript library D3.js v7 [34].

### Database and web interface development

**Fig. 1:**
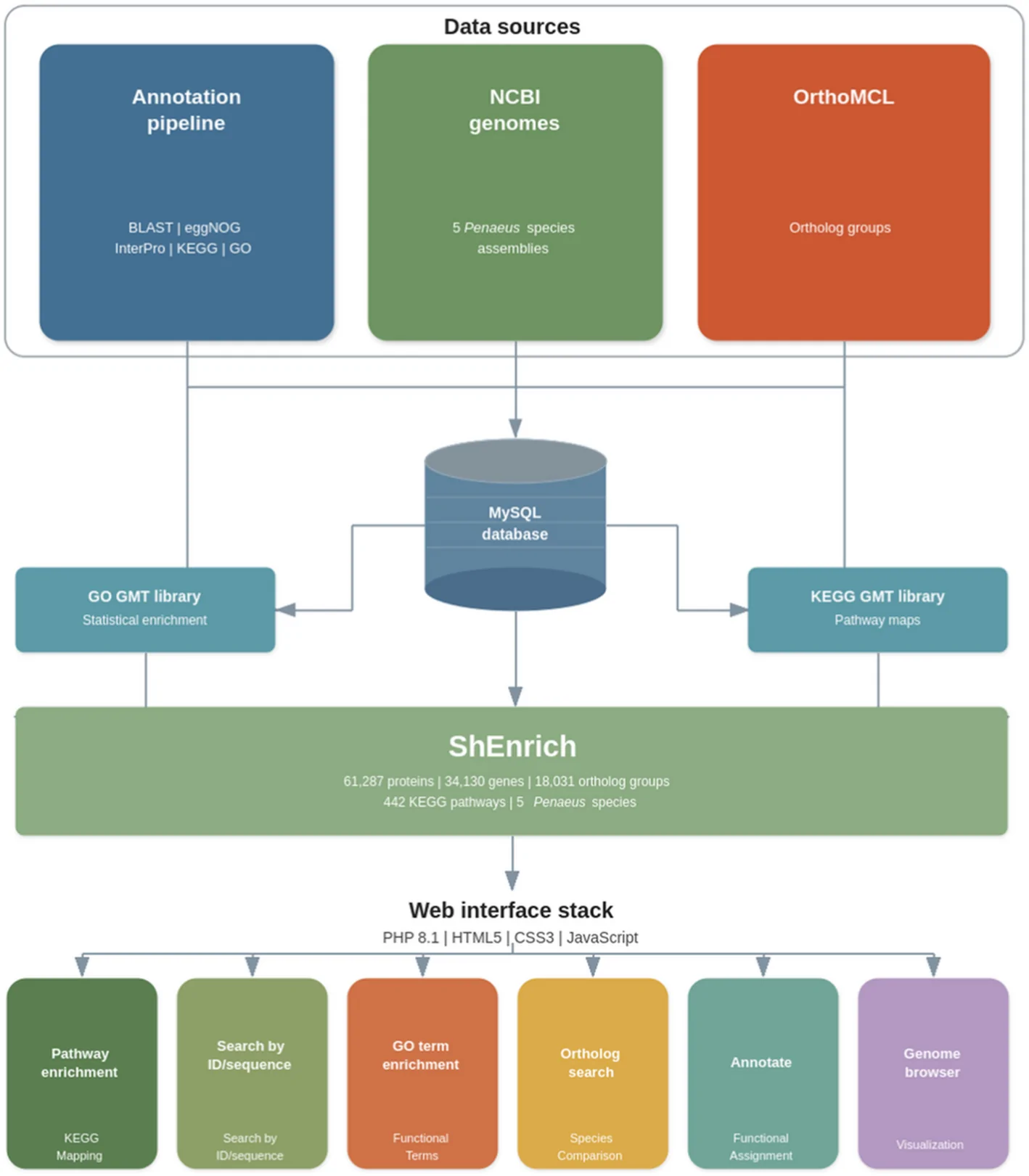
ShEnrich platform workflow showing MySQL 8.0 backend, PHP 8.1 processing layer, and web interface components with relational schema integrating species information, gene annotations, KEGG pathways, GO terms, and ortholog group relationships.

Database (Fig. 1) was developed using the MySQL 8.0 relational database management system (RDBMS), with structured tables designed to store species information, gene annotations, pathway assignments, GO term associations, and ortholog group memberships. The database schema consists of two core tables, a protein annotation table storing functional annotations including GO terms, enzyme codes, and InterPro domain entries, and a gene-pathway-reaction table storing KEGG orthology assignments and pathway associations, linked by protein sequence identifiers, with additional tables supporting ortholog group storage and genomic coordinate retrieval. The web interface was developed using HTML5/CSS3/JavaScript for the frontend and PHP 8.1 for server-side processing. User-uploaded gene lists are processed in memory during the active session and are not written to permanent storage on the server, ensuring that no user data is retained beyond the duration of the active browser session.

### Genome browser integration

To visualize genes on respective shrimp genomes, JBrowse2 (v3.2.0) [35], an embedded genome browser was integrated into ShEnrich for visualization of annotated genes on their respective assemblies. The visualizations include chromosomal positions, orientation, and functional classifications, with interactive navigation from search results to genomic loci.

## Utility and discussion

### Web interface and navigation

**Fig. 2:**
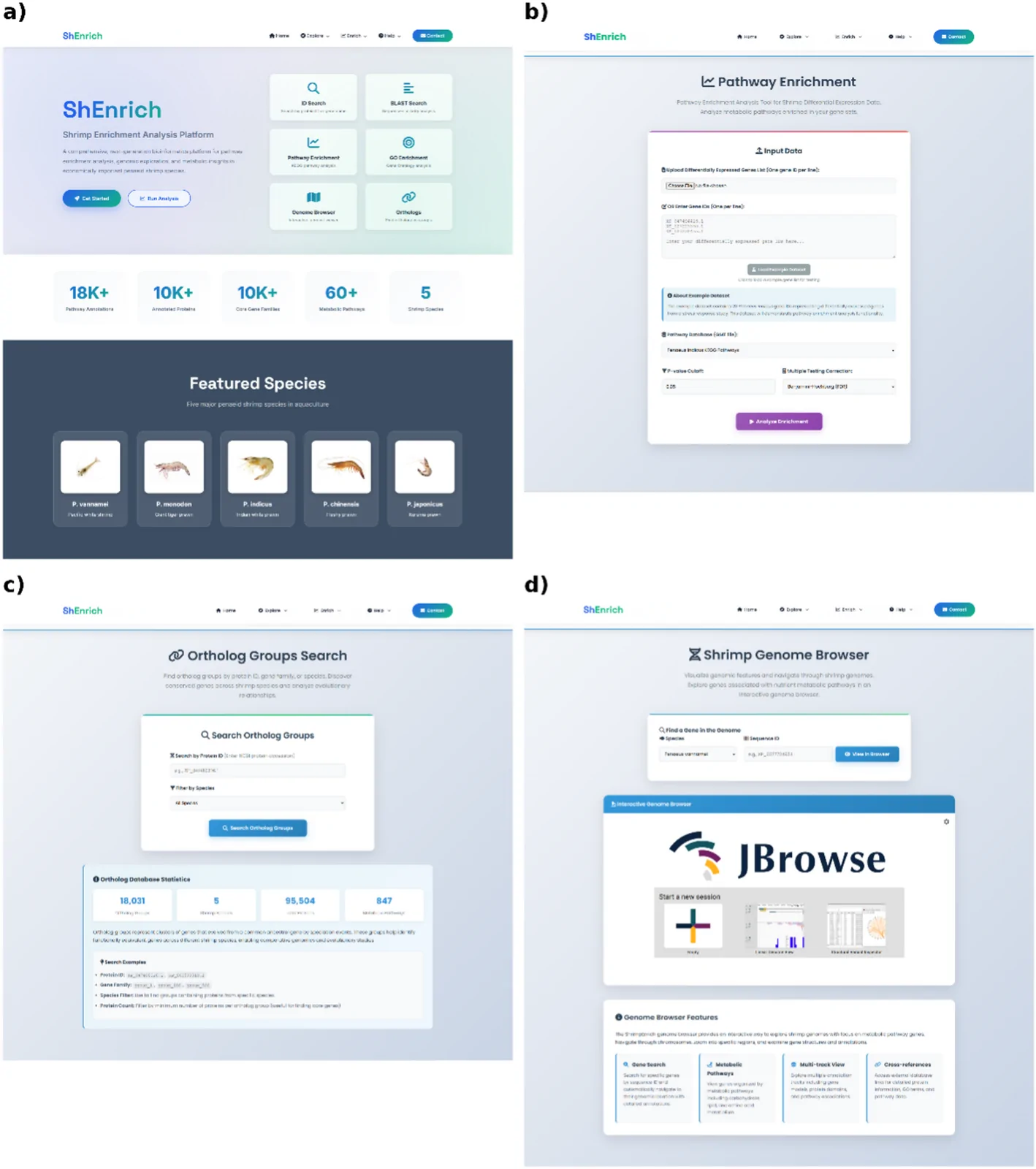
ShEnrich web interface and platform features. **a** ShEnrich homepage showing the platform overview, navigation menus, database statistics, and access points to all analytical features including ID search, BLAST search, pathway enrichment, GO enrichment, genome browser, and ortholog search. **b** Pathway enrichment input interface showing gene list upload options, species-specific GMT library selection, p-value cutoff, and multiple testing correction parameters. **c** Ortholog group search interface showing search options, database statistics, and results panel with species composition and protein member lists. **d** Integrated JBrowse2 (v3.2.0) genome browser interface showing gene navigation by sequence ID, interactive linear genome view, and gene annotation tracks on reference genome assemblies

ShEnrich is freely accessible at https://bioinfo.ciba.res.in/shenrich/. The homepage (Fig. 2a) presents an overview of database contents and direct access to all platform features. The interface is organized into two sections: Explore, which provides tools for browsing and retrieving functional annotations, and Enrich, which provides KEGG pathway and GO term enrichment analysis for user-supplied gene lists. Comprehensive documentation is available through the Help section on the main navigation menu.

### Functional annotation of genes

A primary use of ShEnrich is retrieving functional information for genes identified in transcriptomic experiments. The Search by ID tool accepts protein accession identifiers in XP_ format and returns a consolidated annotation record covering the protein description, KEGG pathway assignments, GO terms across Biological Process, Molecular Function, and Cellular Component ontologies, InterPro domain entries, genomic coordinates, and ortholog group membership. For example, querying XP_027225530.1 from *P. vannamei* returns its complete functional profile including pathway membership, domain architecture, and orthologs across all five species in a single view, removing the need to query multiple databases for a single protein.

For researchers working with sequences that lack standard identifiers, such as those derived from transcriptome assemblies, the Search by Sequence tool performs BLASTP searches against the integrated penaeid shrimp protein datasets and returns annotation records for the best-matching proteins. This extends the utility of ShEnrich to studies that do not use reference genome-based workflows.

The Annotate tool supports large-scale annotation retrieval for gene lists from transcriptomic studies. Users upload a plain text file of protein identifiers or paste them directly into the interface. The tool returns consolidated annotations for all valid inputs along with a summary of annotated, unmatched, and total records. Results are exportable in CSV and TSV formats for direct integration into downstream analysis workflows.

### Pathway and GO term enrichment analysis

ShEnrich performs enrichment analysis through the Enrich menu using GMT libraries built from sequence annotations. Users provide a gene list as protein accession identifiers, select the target species, and configure statistical parameters including p-value threshold and multiple testing correction method. Enrichment analysis is implemented through the clusterProfiler R package v4.17.0 [32], applying Fisher’s exact test for over-representation against the species-specific background gene set. Results are presented in a table showing enriched term identifiers, descriptions, gene counts, enrichment ratios, p-values, and adjusted p-values, with visualization as dot plots, bar charts, and gene-concept network diagrams (Fig. 2b). All outputs are downloadable in TSV and SVG format.

### Cross-species comparative analysis

Understanding whether a gene of interest is conserved across penaeid shrimp species is often critical for interpreting its functional significance. The ortholog search interface accepts a protein identifier from any of the five species and returns the corresponding OrthoMCL group with member counts, species composition, and complete gene lists with links to individual annotation records (Fig. 2c). A researcher who identifies a candidate immunity gene in *P. vannamei*, for instance, can immediately query whether orthologous genes exist in *P. indicus* or *P. monodon* and retrieve their annotations, supporting comparative studies without manual cross-database searches. The 18,031 ortholog groups identified across the five species represent a curated resource for comparative penaeid genomics that was previously unavailable in a unified interface.

### Genomic context visualization

ShEnrich integrates JBrowse2 (v3.2.0) to enable visualization of annotated genes across all five reference genome assemblies (Fig. 2d). Users can navigate to specific chromosomal regions, examine gene structures and orientations, and view associated KEGG and GO annotations directly in the browser. Navigation links from search results and enrichment outputs connect directly to the corresponding genomic coordinates. This allows researchers to move seamlessly from a list of differentially expressed genes to their chromosomal positions, which is particularly useful for identifying gene clusters or co-localizing genes involved in the same biological process.

### Data analysis example

To demonstrate the utility of ShEnrich for functional interpretation of transcriptomic data in penaeid shrimp, we analysed a published *Penaeus vannamei* hepatopancreas RNA-seq dataset examining the response to PirA and PirB toxins, the causative agent of Acute Hepatopancreatic Necrosis Disease ([36]; GEO accession GSE200137). Raw count data from six-hour post-exposure samples and controls were retrieved and differential expression analysis was performed using DESeq2 [37], yielding 1,202 DEGs (|log2FC| > 1, padj < 0.05).

**Fig. 3:**
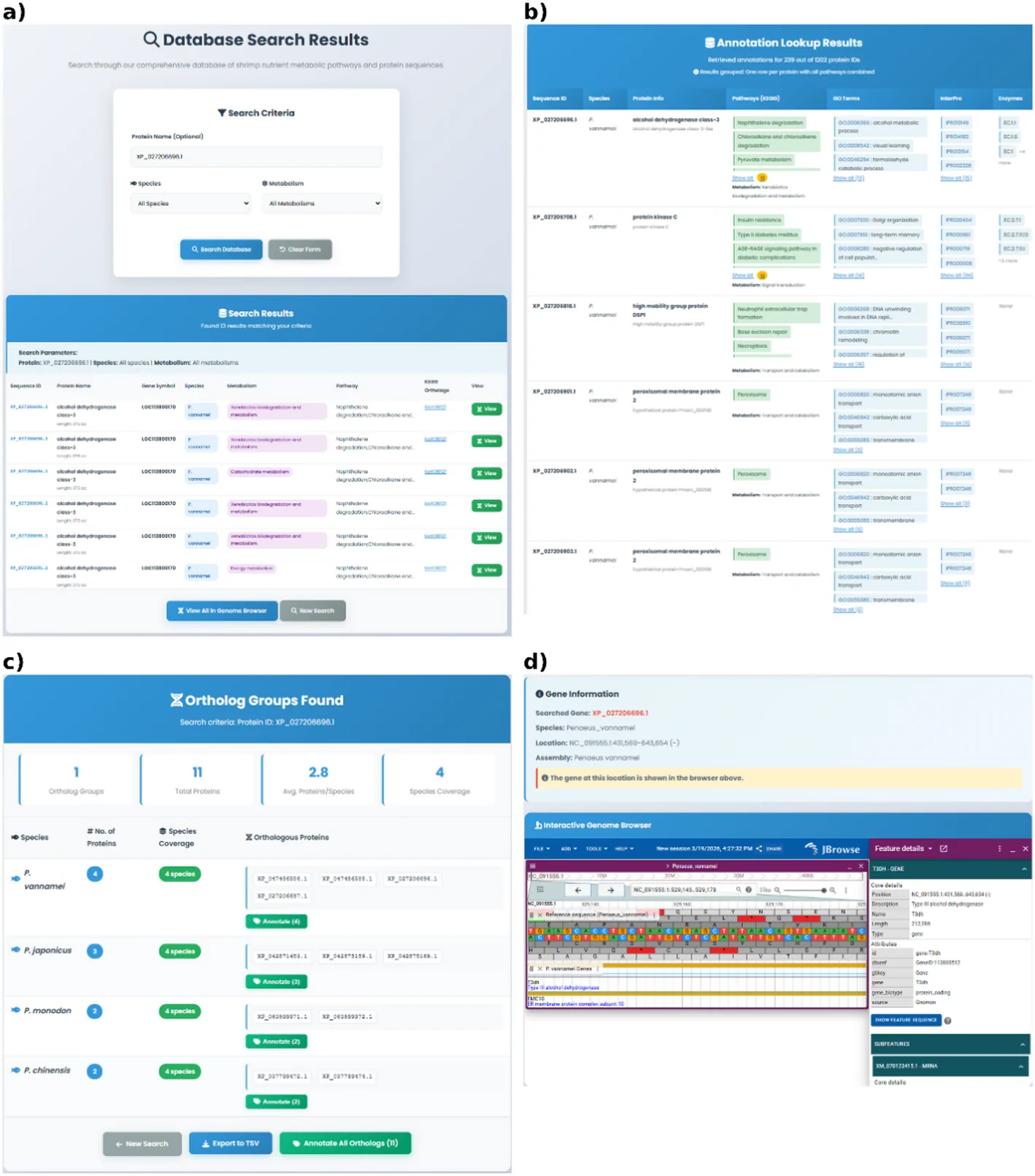
ShEnrich analytical workflow demonstrated using a *Penaeus vannamei* AHPND dataset. **a** Database search result for protein XP_027206696.1 showing annotation records including protein description, KEGG pathway assignments, GO terms, and enzyme classifications. **b** Batch annotation results for 1202 differentially expressed genes showing consolidated functional annotations including protein descriptions, KEGG pathways, GO terms, and InterPro domain entries. **c** Ortholog group search result for XP_027206696.1 showing one ortholog group containing 11 proteins distributed across four species (*P. vannamei*, *P. japonicus*, *P. monodon*, and *P. chinensis*). **d** JBrowse2 genome browser view of XP_027206696.1 showing chromosomal location on NC_091555.1, gene structure, and neighboring loci

Individual genes of interest can be interrogated through the Search by ID tool. Querying XP_027206696.1, a calcium-activated chloride channel regulator identified among the DEGs, returned its complete annotation record including pathway membership and genomic coordinates in a single view (Fig. 3a). The DEG list was submitted to the Annotate tool, which queried the ShEnrich database and returned consolidated functional annotations including protein descriptions, KEGG pathway assignments, GO terms, InterPro domain entries, and enzyme classifications in a single exportable table (Fig. 3b). The ortholog search for this gene identified orthologous sequences distributed across four of the five species in ShEnrich, with the corresponding OrthoMCL group containing 11 proteins across *P. vannamei*, *P. japonicus*, *P. monodon*, and *P. chinensis* (Fig. 3c). Genomic context was visualized through the integrated JBrowse2 browser, which located the gene on chromosome NC_091555.1 and displayed its annotated structure alongside neighboring loci (Fig. 3d).

**Fig. 4:**
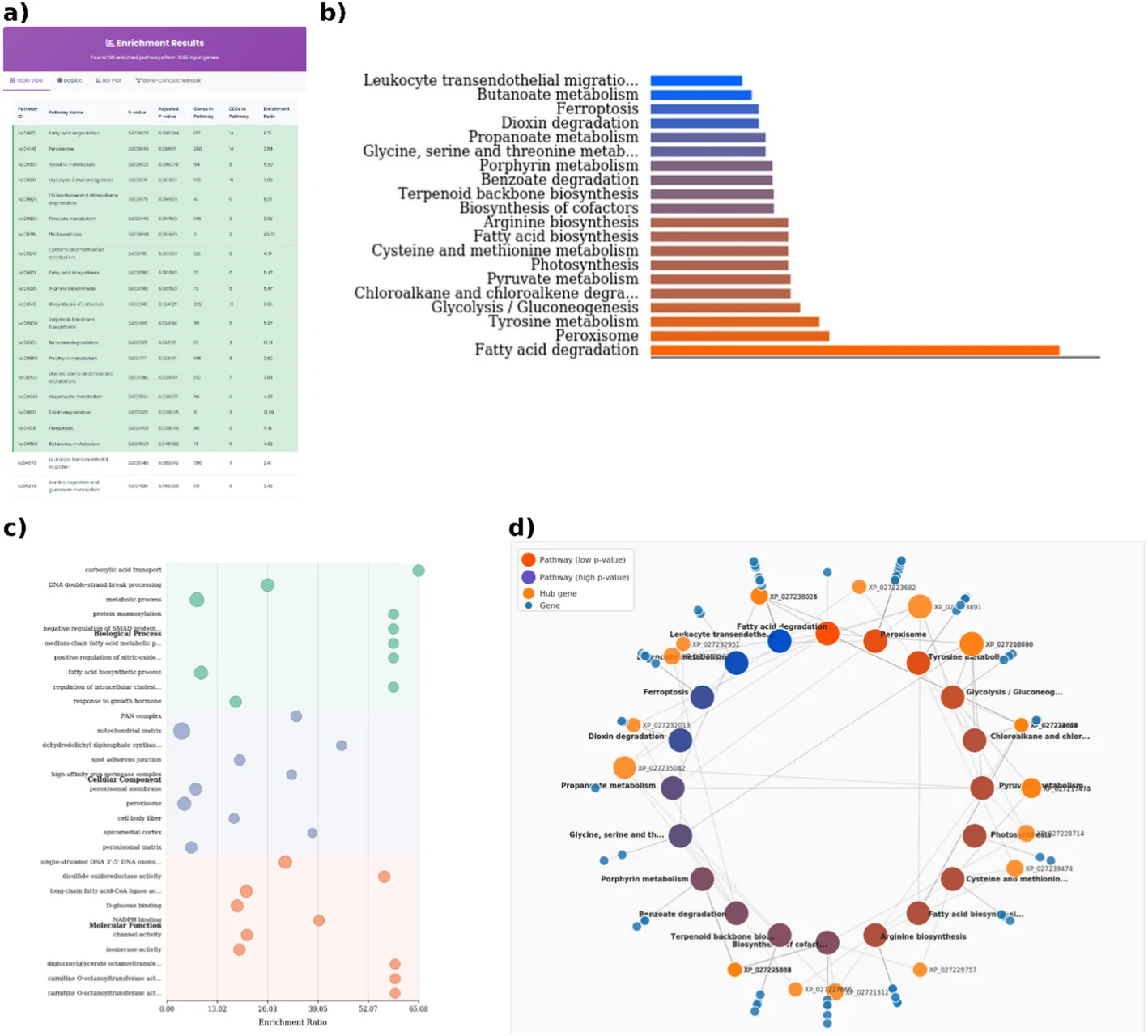
KEGG pathway and GO term enrichment analysis of Penaeus vannamei hepatopancreas differentially expressed genes following PirAB toxin exposure. **a** Enrichment results table showing significantly enriched KEGG pathways with *p*-values, adjusted *p*-values, gene counts, and enrichment ratios. **b** Bar chart of top enriched KEGG pathways ranked by statistical significance, colored by adjusted *p*-value. **c** Combined GO term enrichment dotplot showing significantly enriched terms across Biological Process, Cellular Component, and Molecular Function ontologies, with dot size representing gene count and position representing enrichment ratio. **d** Gene-concept network illustrating bipartite relationships between hub genes and enriched KEGG pathways*.*

KEGG pathway enrichment analysis using the *P. vannamei*-specific GMT library with Benjamini-Hochberg correction identified 198 enriched pathways, of which 19 were significant (padj < 0.05) from the 1,202 input genes (Fig. 4a, b). The most strongly enriched pathway was fatty acid degradation (ko00071; 14 DEGs, fold enrichment = 6.71, padj = 4 × 10⁻^6^), followed by peroxisome (ko04146; fold enrichment = 3.54, padj = 0.005), tyrosine metabolism (ko00350; fold enrichment = 5.59, padj = 0.006), and glycolysis/gluconeogenesis (ko00010; fold enrichment = 3.98, padj = 0.011). GO term enrichment analysis identified significant terms across all three ontologies (Fig. 4c), including carboxylic acid transport (Biological Process; enrichment ratio = 65.08, padj = 2 × 10⁻^6^), mitochondrial matrix (Cellular Component; padj < 0.001), and long-chain fatty acid-CoA ligase activity (Molecular Function; padj < 0.001). The gene-concept network (Fig. 4d) identified hub genes connected to multiple enriched pathways, illustrating shared gene membership across metabolically related processes in the hepatopancreatic response. These results demonstrate ShEnrich’s capability to recover biologically coherent pathway signals from penaeid shrimp transcriptomic data using species-specific GMT libraries, which are not available in generic enrichment tools.

### Comparison with existing tools

*P. indicus*,* P. chinensis*, and *P. japonicus* are entirely absent from DAVID, Enrichr, and pathDIP. Though *P. vannamei* and *P. monodon* are hosted at DAVID, they do not reflect latest annotations. getENRICH was recently developed to support enrichment analysis for non-model organisms through KO-based input [17], and represents a step toward addressing this gap. However, it does not include a species-specific annotation pipeline and does not provide curated gene sets for penaeid shrimp. The SalmoBase database [5] demonstrates what a dedicated annotation resource can provide for an aquaculture group, giving salmonid researchers access to species-appropriate functional data that generic tools cannot offer. ShEnrich provides the equivalent for penaeid shrimp, with the added components of ortholog clustering, genome browser integration, and an easy-to-use annotation pipeline for biologists.

### Discussion

The example use case presented here illustrates that species-specific GMT libraries built from penaeid shrimp annotations return biologically relevant pathway signals from transcriptomic data. For three of the five species covered by ShEnrich (*P. indicus*,* P. chinensis*,* and P. japonicus*), no equivalent enrichment analysis is possible with DAVID, Enrichr, or pathDIP due to the absence of these species from those databases. For *P. vannamei* and *P. monodon*, coverage exists in these tools but is limited by the underrepresentation of crustacean sequences in model organism-centric annotation databases. The primary advantage of ShEnrich lies in the use of species-specific background gene sets derived from each species’ own annotated proteome, which directly determines which pathways appear as significantly enriched. The annotation pipeline is not species-specific in design and can be extended to any organism with a sequenced genome in a public repository, positioning ShEnrich as both an immediate resource for the penaeid research community and a reproducible framework for equivalent resources in other non-model aquaculture species. The annotations are computationally derived and lack experimental validation for most pathway assignments. Completeness varies across species depending on assembly quality [38], and penaeid shrimp genomes are further characterized by high repetitive content and large genome sizes that affect assembly completeness and annotation coverage for some species [7, 18]. Genes with limited similarity to characterized proteins remain difficult to annotate reliably [39]. These are shared limitations of annotation resources for non-model organisms generally [40] and will be addressed as genome assemblies and experimental functional data improve.

ShEnrich will be expanded to include additional crustacean and aquaculture species as high-quality genome assemblies become available. Periodic updates to annotation databases and GMT libraries will be implemented to keep pace with advances in shrimp functional genomics. Support for gene set enrichment analysis using ranked gene lists will be added to complement the current over-representation analysis, providing options for studies that generate continuous expression scores. Integration of experimental expression data and curated pathway annotations as they become available for penaeid shrimp will further improve biological accuracy over time.

## Conclusions

ShEnrich is the first dedicated functional annotation database and enrichment analysis resource for penaeid shrimp, filling a gap that has limited the biological interpretation of transcriptomic data in this important group of aquaculture species. The resource integrates 61,287 annotated proteins representing 34,169 genes across five commercially important species, with 178,595 gene-pathway associations spanning 442 KEGG pathways and 138,077 proteins mapped to 12,897 GO terms. The annotation pipeline, the species-specific GMT libraries and the ortholog groups identified across the five species provide a comparative genomics resource that supports cross-species functional inference. Together, these components establish the functional genomics infrastructure that the penaeid shrimp research community has lacked, and provide a foundation for more accurate interpretation of transcriptomic studies on disease resistance, stress responses, environmental adaptation, and animal physiology in commercially important shrimp species.

## Data Availability

The ShEnrich home page: https://bioinfo.ciba.res.in/shenrich/. The protein datasets used for this study were obtained from genome assemblies available at GenBank: P. vannamei (GCF_042767895.1; ASM4276789v1), P. monodon (GCF_015228065.2; NSTDA_Pmon_1), P. chinensis (GCF_019202785.1; ASM1920278v2), P. indicus (GCF_018983055.1; CIBA_Pind_1.3), and P. japonicus (GCF_017312705.1; Mj_TUMSAT_v1.0).

## References

[1] Food and Agriculture Organization of the United Nations (FAO). The state of world fisheries and aquaculture 2022. Rome: FAO; 2022. 10.4060/cc0461en.

[2] Chicco D, Agapito G. Nine quick tips for pathway enrichment analysis. PLoS Comput Biol. 2022;18:e1010348. 10.1371/journal.pcbi.1010348. PMC9371296 35951505

[3] Rhee SY. The Arabidopsis information resource (TAIR): a model organism database providing a centralized, curated gateway to Arabidopsis biology, research materials and community. Nucleic Acids Res. 2003;31:224–8. 10.1093/nar/gkg076. PMC165523 12519987

[4] Öztürk-Çolak A, Marygold SJ, Antonazzo G, Attrill H, Goutte-Gattat D, Jenkins VK, et al. FlyBase: updates to the *Drosophila* genes and genomes database. Genetics. 2024;227:iyad211. 10.1093/genetics/iyad211. PMC11075543 38301657

[5] Samy JKA, Mulugeta TD, Nome T, Sandve SR, Grammes F, Kent MP, et al. SalmoBase: an integrated molecular data resource for Salmonid species. BMC Genomics. 2017;18:482. 10.1186/s12864-017-3877-1. PMC5485693 28651544

[6] Liu Z, Liu S, Yao J, Bao L, Zhang J, Li Y, et al. The channel catfish genome sequence provides insights into the evolution of scale formation in teleosts. Nat Commun. 2016;7:11757. 10.1038/ncomms11757. PMC4895719 27249958

[7] Zhang X, Yuan J, Sun Y, Li S, Gao Y, Yu Y, et al. Penaeid shrimp genome provides insights into benthic adaptation and frequent molting. Nat Commun. 2019;10:356. 10.1038/s41467-018-08197-4. PMC6341167 30664654

[8] Uengwetwanit T, Pootakham W, Nookaew I, Sonthirod C, Angthong P, Sittikankaew K, et al. A chromosome-level assembly of the black tiger shrimp (*Penaeus monodon*) genome facilitates the identification of growth‐associated genes. Mol Ecol Resour. 2021;21:1620–40. 10.1111/1755-0998.13357. PMC8197738 33586292

[9] Wang Q, Ren X, Liu P, Li J, Lv J, Wang J et al. High-quality genome assembly of Chinese shrimp (*Fenneropenaeus chinensis*) suggests genome contraction and adaptation to the environment. 2021. 10.22541/au.161798993.31069269/v1 34240531

[10] Kawato S, Nishitsuji K, Arimoto A, Hisata K, Kawamitsu M, Nozaki R et al. Genome and transcriptome assemblies of the kuruma shrimp, *Marsupenaeus japonicus*. G3 Genes Genomes Genetics. 2021;11:jkab268. 10.1093/g3journal/jkab268 PMC8527471 34515781

[11] Katneni VK, Shekhar MS, Jangam AK, Krishnan K, Prabhudas SK, Kaikkolante N, et al. A superior contiguous whole genome assembly for shrimp (*Penaeus indicus*). Front Mar Sci. 2022;8:808354. 10.3389/fmars.2021.808354.

[12] Krishnan K, Prabhudas SK, Jayaraman K, Angel JRJ, Jangam AK, Katneni VK, et al. Transcriptomic variations associated with salinity stress in *Penaeus indicus*. Mol Biol Rep. 2023;50:9295–306. 10.1007/s11033-023-08824-4. 37812353

[13] Palliyath GK, Jangam AK, Katneni VK, Kaikkolante N, Panjan Nathamuni S, Jayaraman R, et al. Meta-analysis to unravel core transcriptomic responses in *Penaeus vannamei* exposed to biotic and abiotic stresses. Biochem Genet. 2025;63:1459–78. 10.1007/s10528-024-10772-y. 38570440

[14] Huang DW, Sherman BT, Lempicki RA. Systematic and integrative analysis of large gene lists using DAVID bioinformatics resources. Nat Protoc. 2009;4:44–57. 10.1038/nprot.2008.211. 19131956

[15] Chen EY, Tan CM, Kou Y, Duan Q, Wang Z, Meirelles GV, et al. Enrichr: interactive and collaborative HTML5 gene list enrichment analysis tool. BMC Bioinformatics. 2013;14:128. 10.1186/1471-2105-14-128. PMC3637064 23586463

[16] Rahmati S, Abovsky M, Pastrello C, Jurisica I. pathDIP: an annotated resource for known and predicted human gene-pathway associations and pathway enrichment analysis. Nucleic Acids Res. 2017;45:D419–26. 10.1093/nar/gkw1082. PMC5210562 27899558

[17] Bhatia A, Pruthi P, Chakraborty I, Shukla N, Narayan J. getENRICH: a tool for the gene and pathway enrichment analysis of non-model organisms. Bioinf Adv. 2024;5:vbaf023. 10.1093/bioadv/vbaf023. PMC11845281 39990255

[18] Yuan J, Zhang X, Li F, Xiang J. Genome sequencing and assembly strategies and a comparative analysis of the genomic characteristics in penaeid shrimp species. Front Genet. 2021;12:658619. 10.3389/fgene.2021.658619. PMC8126689 34012463

[19] Vijayan KK. Domestication and genetic improvement of Indian white shrimp, penaeus indicus: a complimentary native option to exotic pacific white shrimp, *Penaeus vannamei*. J Coastal Res. 2019;86:270. 10.2112/SI86-040.1.

[20] Ekblom R, Wolf JBW. A field guide to whole-genome sequencing, assembly and annotation. Evol Appl. 2014;7:1026–42. 10.1111/eva.12178. PMC4231593 25553065

[21] Bolger ME, Arsova B, Usadel B. Plant genome and transcriptome annotations: from misconceptions to simple solutions. Brief Bioinform. 2017;bbw135. 10.1093/bib/bbw135. PMC5952960 28062412

[22] Liao J, Lai Z, He C, Li G, Dong Z, Guo Y, et al. A high-quality chromosome-level genome assembly of Pacific whiteleg shrimp (*Penaeus vannamei*). Sci Data. 2025;12:340. 10.1038/s41597-025-04673-x. PMC11865283 40011501

[23] Altschul S. Gapped BLAST and PSI-BLAST: a new generation of protein database search programs. Nucleic Acids Res. 1997;25:3389–402. 10.1093/nar/25.17.3389. PMC146917 9254694

[24] The Gene Ontology Consortium, Carbon S, Douglass E, Good BM, Unni DR, Harris NL, et al. The gene ontology resource: enriching a GOld mine. Nucleic Acids Res. 2021;49:D325–34. 10.1093/nar/gkaa1113. PMC7779012 33290552

[25] Jones P, Binns D, Chang H-Y, Fraser M, Li W, McAnulla C, et al. InterProScan 5: genome-scale protein function classification. Bioinformatics. 2014;30:1236–40. 10.1093/bioinformatics/btu031. PMC3998142 24451626

[26] Huerta-Cepas J, Szklarczyk D, Heller D, Hernández-Plaza A, Forslund SK, Cook H, et al. eggNOG 5.0: a hierarchical, functionally and phylogenetically annotated orthology resource based on 5090 organisms and 2502 viruses. Nucleic Acids Res. 2019;47:D309–14. 10.1093/nar/gky1085. PMC6324079 30418610

[27] Götz S, García-Gómez JM, Terol J, Williams TD, Nagaraj SH, Nueda MJ, et al. High-throughput functional annotation and data mining with the Blast2GO suite. Nucleic Acids Res. 2008;36:3420–35. 10.1093/nar/gkn176 PMC2425479 18445632

[28] Li L, Stoeckert CJ, Roos DS. OrthoMCL: identification of ortholog groups for eukaryotic genomes. Genome Res. 2003;13:2178–89. 10.1101/gr.1224503. PMC403725 12952885

[29] van Dongen SM. Graph clustering by flow simulation. PhD thesis. Utrecht University; 2000.

[30] Subramanian A, Tamayo P, Mootha VK, Mukherjee S, Ebert BL, Gillette MA, et al. Gene set enrichment analysis: a knowledge-based approach for interpreting genome-wide expression profiles. Proc Natl Acad Sci USA. 2005;102:15545–50. 10.1073/pnas.0506580102. PMC1239896 16199517

[31] Yu G, Wang L-G, Han Y, He Q-Y. clusterProfiler: an R package for comparing biological themes among gene clusters. OMICS. 2012;16:284–7. 10.1089/omi.2011.0118. PMC3339379 22455463

[32] Wu T, Hu E, Xu S, Chen M, Guo P, Dai Z, et al. clusterProfiler 4.0: a universal enrichment tool for interpreting omics data. Innov. 2021;2:100141. 10.1016/j.xinn.2021.100141. PMC8454663 34557778

[33] Benjamini Y, Hochberg Y. Controlling the false discovery rate: a practical and powerful approach to multiple testing. J Royal Stat Soc Ser B Stat Methodol. 1995;57:289–300. 10.1111/j.2517-6161.1995.tb02031.x.

[34] Bostock M, Ogievetsky V, Heer J. D^3^ data-driven documents. IEEE Trans Visual Comput Graphics. 2011;17:2301–9. 10.1109/TVCG.2011.185. 22034350

[35] Diesh C, Stevens GJ, Xie P, De Jesus Martinez T, Hershberg EA, Leung A, et al. JBrowse 2: a modular genome browser with views of synteny and structural variation. Genome Biol. 2023;24:74. 10.1186/s13059-023-02914-z. PMC10108523 37069644

[36] Lange MD, Abernathy J, Rawles AA, Zhang D, Shoemaker CA, Bader TJ, et al. Transcriptome analysis of Pacific white shrimp (*Liptopenaeus vannamei*) after exposure to recombinant Vibrio parahaemolyticus PirA and PirB proteins. Fish Shellfish Immunol. 2023;132:108502. 10.1016/j.fsi.2022.108502. 36565998

[37] Love MI, Huber W, Anders S. Moderated estimation of fold change and dispersion for RNA-seq data with DESeq2. Genome Biol. 2014;15:550. 10.1186/s13059-014-0550-8. PMC4302049 25516281

[38] Salzberg SL, Yorke JA. Beware of mis-assembled genomes. Bioinformatics. 2005;21:4320–1. 10.1093/bioinformatics/bti769. 16332717

[39] Yandell M, Ence D. A beginner’s guide to eukaryotic genome annotation. Nat Rev Genet. 2012;13:329–42. 10.1038/nrg3174. 22510764

[40] Shekhar MS, Katneni VK, Jangam AK, Krishnan K, Kaikkolante N, Vijayan KK. The genomics of the farmed shrimp: current status and application. Rev Fish Sci Aquacult. 2021;29:654–65. 10.1080/23308249.2020.1858271.

